# Reduction in Arterial Stiffness Index (SI) in Response to Combination Antioxidant Therapy

**DOI:** 10.3390/jcm12216804

**Published:** 2023-10-27

**Authors:** Laurence Guy Howes, Tanya Unni, Ameer Hamza, Jan B. Howes, Rohan Jayasinghe

**Affiliations:** 1 Department of Cardiology, Gold Coast University Hospital, Gold Coast 4215, Australia; rohan.jayasinghe@health.qld.gov.au; 2 School of Medicine, Griffith University, Gold Coast 4215, Australia; 3 School of Medicine, Bond University, Gold Coast 4227, Australia; 4 Amtan Medical Centers, Gold Coast 4208, Australia; tanya@amtanmedical.com.au (T.U.); ameer@amtanmedical.com.au (A.H.); 5 Phoenix Pharmaceuticals Australia Pty Ltd., Sanctuary Cove, Gold Coast 4212, Australia; janhowes@05acl.com.au

**Keywords:** antioxidants, arterial stiffness, pulse wave velocity, stiffness index

## Abstract

Antioxidants reduce arterial stiffness, but the effects previously reported are weak. A systematic review of the antioxidants vitamin E, vitamin C, vitamin A, and beta-carotenes (the most commonly studied antioxidants) on pulse wave velocity (PWV) found an effect size of only −0.20 (approximately −16 m/s or −2.5%). Studies in rats of the potent pro-oxidant substance acetaldehyde have shown that combinations of sulfur-containing antioxidants, including thiamine and l-cysteine, with ascorbic acid potently protect against oxidative-stress-mediated mortality. The effects of these combinations of oxidants on PWV have not been studied. The present study evaluated the effects of 2 weeks of therapy with a combination of sulfur-containing antioxidants (cysteine, thiamine, and pyridoxine) in combination with ascorbic acid on stiffness index (SI), a measure of arterial stiffness that is strongly correlated with PWV, using a Pulse Trace recorder in a diverse group of 78 volunteers. SI fell by −1.7 m/s relative to placebo (95% confidence intervals −0.6 to −2.7 m/s), a reduction of −19% (95% confidence intervals −9% to −31%). The Glass effect size was 1.4, indicating a very strong treatment effect which was substantially greater than the effect size found in previous studies of antioxidants. PWV reduction was correlated significantly with increasing age. Further studies of similar antioxidant combinations are required to determine whether they are of value in the treatment or prevention of cardiovascular disease.

## 1. Introduction

Arterial stiffness, generally measured using direct or indirect measurements of pulse wave velocity (PWV), is a strong risk factor for major adverse cardiovascular events (MACE) [1]. Oxidative stress is an important contributor to arterial stiffness [2]. However, studies aiming to use antioxidant therapy to reduce PWV have generally found only small reductions in PWV of approximately −0.16 m/s or −2.5% [3,4]. Most studies have investigated the effects of only one or two different antioxidants [3]. Multiple-antioxidant therapy has been reported to improve cardiovascular outcomes compared to monotherapy in animal models. The effects have been reported to include changes in Endothelin and Galectin-3 [5,6]. Studies of the potent pro-oxidant substance acetaldehyde in rats have shown that combinations of sulfur-containing antioxidants, including thiamine and l-cysteine, with ascorbic acid potently protect against oxidative-stress-mediated mortality [7]. However, the effects of these compounds on pulse PWV have not been studied.

The present study measured changes in the arterial stiffness index (SI—a measure of PWV) using pulse wave analysis following two weeks of treatment using an oral formulation of four antioxidants (l-cysteine, thiamine, pyridoxine, and ascorbic acid) compared to placebo. The ingredients and the amounts of antioxidants chosen for the present study were chosen because they provide a diversity of antioxidants, and because the formulation has previously been reported to significantly reduce hangover symptoms [8] which are believed to be largely due to oxidative stress [9].

The demonstration that the combination of antioxidants studied reduced PWV could provide a rationale for studying the combination in the treatment of vascular diseases.

## 2. Methods

### 2.1. Study Design and Subjects

The study followed a single blind, randomized, parallel group design of two weeks of treatment with either the antioxidant formulation or placebo in a diverse group of subjects. A single blind design was used because of difficulty obtaining matching placebos and because the outcomes assessed were measured using electronic devices which avoid the possibility of observer bias. The intention was to study 100 subjects with 50 subjects randomized to each treatment arm. The exclusion criteria were the presence of atrial fibrillation or medication with antioxidant supplements. A diverse group of subjects representative of the general population was chosen so that the results would be of relevance to all groups that may choose to take antioxidant supplements. Subjects were recruited by word of mouth, from patients attending a general practice clinic, and from social groups at housing estates.

### 2.2. Study Procedures

The subjects attended on the first occasion to sign a consent form and for the recording of demographic data and vital signs. Baseline recordings of SI were performed after resting in a seated position for 10 min. The subjects were then given a bottle containing greater-than-two-week supply of the antioxidant formulation or placebo according to a randomization schedule. The schedule was created by an independent pharmacist who sealed the randomization schedule in an envelope that remained sealed until the data collection had been completed. Randomization was performed in blocks of four. The subjects were instructed not to change their medication and to maintain their usual diet, caffeine and alcohol intake and to continue their usual level of physical activity until the study was completed.

The subjects were instructed to take their study medication in the morning at the same time each day and to attend two weeks after the first visit at the same time and same day of the week as the first visit. At the second visit, recordings of SI were again performed approximately 4 h after taking their medication. Vital signs were recorded and the subjects were questioned about the occurrence of possible adverse events. Their bottles of study medication were returned, and the remaining tablets counted to determine compliance. Each subject then received bottles of the antioxidant formulation for open-label treatment to provide additional information on tolerability.

### 2.3. Study Formulations

The antioxidant treatment consisted of two tablets each containing l-cysteine hydrochloride monohydrate 322 mg, thiamine hydrochloride (vitamin B1) 80 mg, pyridoxine hydrochloride (vitamin B6) 10 mg, and ascorbic acid (vitamin C) 150 mg (Bluegum Pharmaceuticals, Botany, NSW, Australia). The tablets were maintained at below 25 degrees Celsius. The formulation underwent regular testing to determine purity and stability.

Placebo capsules were filled with corn flour by an independent pharmacist who also filled dark brown glass bottles with the antioxidant and placebo formulations and labelled the bottles with numbers according to the randomization schedule. As far as was possible, volunteers were kept from conversing with each other to avoid discussions about the appearance of the formulations they were receiving.

### 2.4. Measurement of SI

SI was measured in the sitting position after 10 min of rest using a Pulse Trace recorder (Micro Medical, Gillingham, Kent, UK). Six initial recordings were made to ensure that a stable, good-quality arterial waveform was displayed on the monitor, after which three further recordings were made and the mean values of SI from these three recordings were entered as the baseline and outcome measurements. Patients in whom the Pulse Trace recorder could not calculate SI results (usually because of the absence of a recognizable reflected pulse wave) could not be included in the efficacy analysis but were included in the safety assessment.

**Figure 1:**
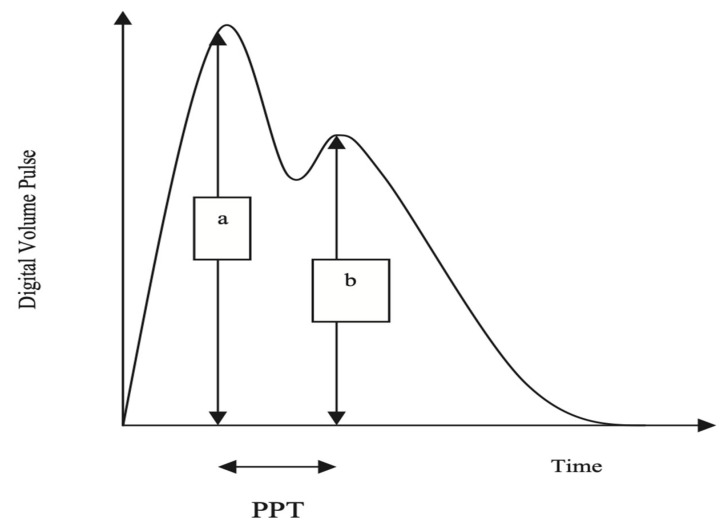
The arterial pulse waveform.

The Pulse Trace recorder uses a digital photoplethysmograph transmitting infrared light technique from a finger cuff applied to the left index finger. The amount of light transmitted through the finger varies proportionally to changes in its blood volume. The signal from the photoplethysmograph obtained over a 30 s period is averaged by the system, to produce a single digital volume pulse (DVP) waveform (Figure 1). Chowienczyk et al. [10] demonstrated that the peripheral pressure pulse is related to the DVP by a single generalized transfer function, which is inbuilt in the Pulse Trace system.

The DVP wave (Figure 1) consists of an early systolic peak (a), which results from an increase in digital blood volume from a pressure wave transmitted from the left ventricle to the finger along a direct path. The second peak, (b), occurs in diastole, and is formed by pressure waves reflected up to the aorta and thence to the finger, from sites of impedance mismatch in the lower body. The time between the systolic and diastolic peaks (peak to peak time, PPT) can be used to infer the time taken for the pressure wave to travel from the aorta to the lower body, and thence as a reflected wave back up to the aorta to the finger. This path length is unknown but is proportional to the subject’s height (h). An index of large arterial stiffness (stiffness index, SI) can therefore be derived; for instance, the calculation of pulse wave velocity (PWV) by the formula h/PPT. SI has been shown to be strongly correlated to central (aortic and carotid femoral) PWV [10].

A previous study in 115 subjects reported good intra-individual reproducibility, with a co-efficient of variation (CV) for SI of 8% [11].

### 2.5. Statistical Analysis

All results are expressed as the mean and standard deviation.

The change in SI, blood pressures, and heart rate from baseline following the two-week treatment period was calculated. Differences between antioxidant and placebo treatments for change from baseline were analyzed using Student’s *t* test. Regression analysis with analysis of variance produced the same results as analysis using Student’s *t* test.

Estimates of the effect size were made according to the method of Cohen (difference between means divided by the pooled standard deviation) [12], or if there was a substantial difference between the standard deviation of the change from baseline between antioxidant and placebo therapy, using the method of Glass (difference between the means divided by the standard deviation of the control group) [13,14]. Effect sizes of 0.3 or less are weak, those greater than 0.8 are strong, and those greater than 1.2 are very strong. Predictors of change in SI were analyzed using linear regression (age, gender, body mass index, SBP, and heart rate).

Baseline differences between the two treatment groups were analyzed using Student’s *t* test or the Chi square test for categoric variables.

The number of subjects required to detect a difference in SI between baseline and the end of the 2-week treatment period of 1.0 m/s between antioxidant and placebo groups (with a power of 80%, assumed equal standard deviations of 1.6 m/s and alpha = 0.05 two tailed) was calculated to be 42 subjects in each group. A standard deviation of 1.60 was calculated from previously collected data, and 1.00 m/s was the smallest change from baseline in SI between groups considered to be clinically significant. A sample size of 50 subjects in each group was chosen to allow for dropouts and failure to obtain arterial waveforms suitable for analysis.

### 2.6. Ethics

The subjects signed a consent form before any research activities commenced. Study conformed with the declaration of Helsinki and the protocol was approved by the Gold Coast University Hospital Ethics Committee.

## 3. Results

**Figure 2:**
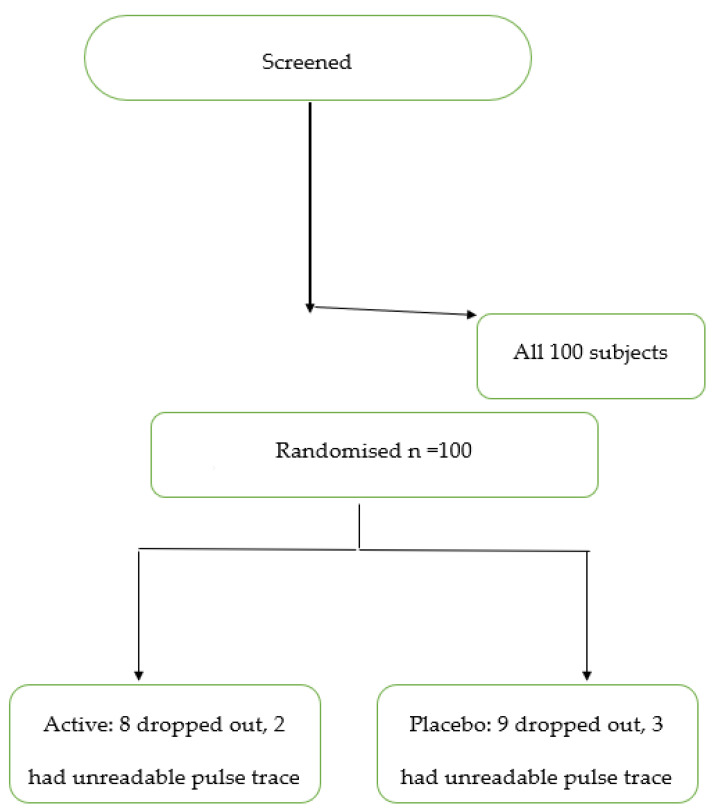
Consort diagram. Active = antioxidant therapy.

One hundred subjects were enrolled in the study and randomized to antioxidant or placebo therapy. Fifteen patients failed to attend for the second study day and 7 subjects had arterial waveforms that could not be analyzed. A total of 40 subjects who were randomized to the antioxidant treatment and 38 patients randomized to placebo had complete data sets. A Consort flow chart is presented in Figure 2.

The baseline characteristics and the changes from baseline to the end of the study of SI, blood pressures, and heart rates are presented in Table 1. The antioxidant and placebo groups were well matched, apart from BMI.

**Figure 3:**
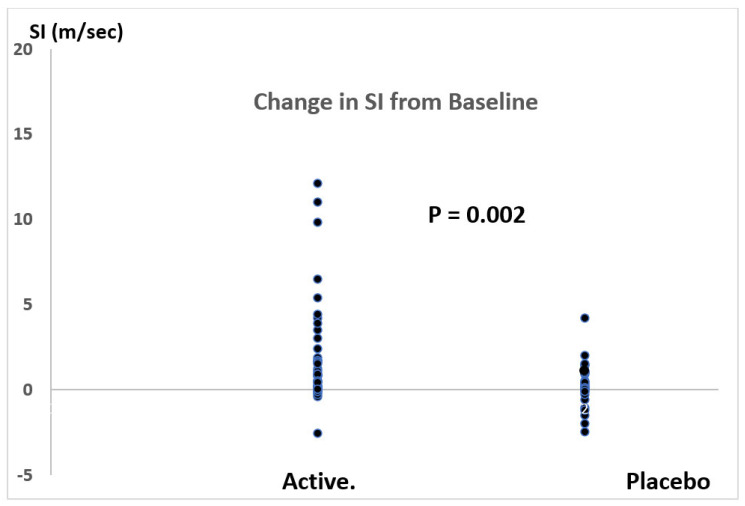
Change in SI following two weeks of antioxidant (active) therapy compared with placebo. *p* = 0.002 (Student’s *t* test).

Changes in PWV from baseline in response to antioxidant and placebo therapy are presented in Figure 3. Antioxidant therapy significantly reduced SI by −1.7 m/s (placebo corrected) with 95% confidence levels of −0.6 to −2.7 m/s. This represented a −19% reduction in SI with 95% confidence levels of −9% to −31%. The effect size for the fall in SI, calculated by the method of Glass (because of unequal variances between antioxidant and placebo therapies), was 1.4.

SI was related to age (r = 0.362, *p* = 0.02). There were no other significant predictors of change in SI and there were no significant differences between antioxidant therapy and placebo on changes in blood pressure or heart rate.

Compliance with medication was greater than 80% in all patients, and there were no reported adverse events.

## 4. Discussion

The combination therapy of four antioxidants reduced SI by −1.7 m/s (placebo-corrected) with an effect size of 1.4 using the method of Glass [13], indicating a very strong effect in reducing PWV. The placebo-corrected percent fall in SI (−19% with 95% confidence limits of −7% and −31%) was impressive. This is a substantially greater reduction in PWV than has been reported for antioxidant therapies in previous studies [3,4]. A meta-analysis of these studies found only a small improvement with antioxidant therapy, with a weak effect size of 0.20 (approximately −0.16 m/s or −2.5%) [3].

The beneficial effects of antioxidant therapies on PWV may be explained by the reduction in the damaging effects of free radicals on the structural and functional components of the vessel walls. Antioxidants inactivate free radicals, reduce inflammation, and, therefore, protect the integrity of the vascular wall [2,3,15,16].

The results of the present study suggest that the antioxidant therapy used may be of value in the management of cardiovascular diseases. However, reductions in PWV during antioxidant therapy cannot be assumed to lead to a reduction in MACE. Studies of the effects of antioxidant supplementation in improving clinical outcomes have been disappointing [17]. Outcome studies assessing the impact of potent antioxidant therapy with similar compositions to that used in the present study are needed.

We studied a diverse population and found that, while the overall effect on PWV was statistically significant and of a substantial magnitude, the variability of PWV responses was greater in the subjects that received antioxidants than in the subjects that received placebo. This suggests that there are individuals or groups that respond to antioxidant therapy more than others. Any benefits in reduction in MACE that may occur with the formulation used in the present study may depend upon the characteristics of the population that was treated. The meta-analysis of previous studies of the effects of antioxidants on PWV found that the greatest effects were in younger women who were not taking additional antioxidants from other sources [3]. The present study found a significant relationship between the subjects’ age and the magnitude of change in SI during antioxidant therapy. Older subjects had a greater fall in SI. Further studies in population subgroups are needed to identify which populations are likely to respond the most to treatment and to perhaps experience the greatest clinical benefit.

The optimal duration of treatment with antioxidants to achieve improvements in PWV and the duration of these effects could not be established from the present study. The meta-analysis of previous studies of antioxidants found that the reduction in PWV was similar during short-term and long-term therapy [3]. The present study aimed to assess the efficacy of combining several antioxidants, including sulfur-containing antioxidants. The study could not define the relative importance of the individual antioxidants on the results achieved, and it is possible that most of the potentially beneficial effects were predominantly due to only one or two of the ingredients. Further studies of the individual components are required to clarify this matter.

The results of the present study compare favorably with studies of therapies other than antioxidants on PWV. Several antihypertensive drugs, including ACE inhibitors and calcium channel blockers, reduce PWV with an effect size of around 0.66 [18]. However, it possible that a considerable portion of the reduction in PWV is secondary to the fall in BP associated with antihypertensive therapy, as PWV has been reported to be closely related to SBP [19]. Nitrates reduce PWV and SBP to a similar extent to antihypertensives [20]. A meta-analysis of the effects of statins found a reduction in PWV with an effect size of 0.37 [21]. Topical, but not oral, estrogens have been reported to reduce PWV with an effect size of approximately 0.33 [22]. A meta-analysis of studies of the effects of isoflavones, which have antioxidant activity, found a reduction in PWV with an effect size of 0.38 [23]. Weight loss of 8% of body weight was accompanied by a fall in PWV, with an effect size of 0.32 [24]. All these reported effect sizes are weak compared to the effect size for the change in SI in the present study.

It has been estimated that for each increase in PWV of 1.0 m/s, there is an increase in MACE of approximately 15% [25]. If an intervention that lowers PWV by 1.0 m/s reduces MACE by about 15%, the 1.7 m/s reduction in PWV achieved in the present study suggests that therapy with the antioxidant formulation that we used may be of value in clinical practice.

The single-blinded design of the present study and the use of placebo, which was different in appearance to the antioxidant formulation, may be a possible limitation of the study. However, the major parameters of interest (SI, BPs, and heart rate) were all calculated by electronic devices which protect against observer bias.

We did not obtain information about the subjects’ medical conditions or drug therapy to investigate whether these factors influenced the effect of the antioxidant therapy on SI. An analysis of the effects of medical disorders and therapy was beyond the scope of the present study, which was intended to determine the effects of the antioxidant therapy on SI in a broad cross section of the community.

In conclusion, supplementation with the combination of antioxidants used in this study improves SI to an extent that may be of therapeutic value. Further studies in patients with a high risk of MACE should now be performed.

## Figures and Tables

**Table 1:** Baseline characteristics and change from baseline at the second study day. Values are the mean ± standard deviation. *p* value for comparisons between the antioxidant and placebo therapies Student’s *t* test.

	Antioxidant n = 40	Placebo n = 38	*p*
Gender:			
Male	40%	34%	-
Female	60%	66%	
Age (years)	62 ± 13	63 ± 10	0.83
SBP (mmHg)	138 ± 21	136 ± 14	0.62
Change in SBP (mmHg)	−1 ± 17	2 ± 10	0.27
DBP (mmHg)	81 ± 11	83 ± 10	0.45
Change in DBP (mmHg)	−2 ± 9	1 ± 9	0.20
Heart rate (bpm)	74 ± 11	73 ± 11	0.79
Change in heart rate (bpm)	−1 ± 6	−1 ± 9	0.88
BMI (kg/m^2^)	27 ± 4	30 ± 6	0.02
SI (m/s)	8.97 ± 3.20	8.78 ± 3.73	0.81
Change in SI (m/s)	−1.95 ± 3.20	−0.30 ± 1.14	0.002

## Data Availability

Data are available from L.G.H.
